# Handheld Ground-Penetrating Radar Antenna Position Estimation Using Factor Graphs

**DOI:** 10.3390/s25113275

**Published:** 2025-05-23

**Authors:** Paweł Słowak, Tomasz Kraszewski, Piotr Kaniewski

**Affiliations:** Faculty of Electronics, Military University of Technology, ul. gen. S. Kaliskiego 2, 00-908 Warsaw, Poland; pawel.slowak@wat.edu.pl (P.S.); tomasz.kraszewski@wat.edu.pl (T.K.)

**Keywords:** factor graph, estimation, ground penetrating radar, ultrawideband radio

## Abstract

Accurate localization of handheld ground-penetrating radar (HH-GPR) systems is critical for high-quality subsurface imaging and precise geospatial mapping of detected buried objects. In our previous works, we demonstrated that a UWB positioning system with an extended Kalman filter (EKF) employing a proprietary pendulum (PND) dynamics model yielded highly accurate results. Building on that foundation, we present a factor-graph-based estimation algorithm to further enhance the accuracy of HH-GPR antenna trajectory estimation. The system was modeled under realistic conditions, and both the EKF and various factor-graph algorithms were implemented. Comparative evaluation indicates that the factor-graph approach achieves an improvement in localization accuracy from over 30 to almost 50 percent compared to the EKF PND. The sparse matrix representation inherent in the factor graph enabled an efficient iterative solution of the underlying linearized system. This enhanced positioning accuracy is expected to facilitate the generation of clearer, more distinct underground images, thereby supporting the potential for more reliable identification and classification of buried objects and infrastructure.

## 1. Introduction

Modern radar-based sensing systems play a crucial role in subsurface exploration, enabling the detection of buried objects and the characterization of underground structures. Such specialized radar systems are commonly called ground-penetrating radars (GPRs). A variety of GPR solutions are available, catering to a wide range of applications in both civilian and military domains [1,2,3,4,5,6]. While GPR systems are frequently deployed on manned or unmanned mobile ground platforms, in challenging environments—such as indoor settings, debris-strewn areas, or densely vegetated terrain—compact and portable handheld ground-penetrating radars (HH-GPRs) are often utilized [7,8,9].

This paper addresses an important problem encountered when using HH-GPR, namely, accurately determining the antenna’s position during scanning. Precise positioning is crucial for correctly mapping the acquired radargrams to the antenna’s successive locations along the non-rectilinear scanning path at the time of signal transmission. Such accurate localization is essential for ensuring reliable geospatial mapping of the data and for enabling effective synthesis of images of the buried objects. However, estimating the trajectory of a handheld GPR antenna is challenging due to factors such as operator-induced motion, terrain irregularities, and external disturbances affecting sensor measurements [10,11].

Traditional localization methods, such as the Kalman filter (KF) [12,13], rely on the assumption of a Markov process, where each estimated state is dependent only on its immediate predecessor. The KF employs recursive updates to estimate the system’s state based on sequential measurements, making it computationally efficient [12,13,14]. However, its accuracy is often limited by the inability to fully incorporate complex motion constraints and measurement uncertainties within each iteration. An alternative approach to solving the localization problem is the use of factor graphs, in which nodes represent the estimated positions of the radar system at different time steps, and edges encode constraints between these positions. Factor graphs offer a batch optimization framework for trajectory estimation by jointly considering all available measurements and state transitions, as opposed to the sequential updates used in filtering techniques. This smoothing-based approach allows for improved handling of nonlinear motion models and sparse or noisy observations.

Originally introduced by Kschischang et al. [15] for function factorization in probabilistic graphical models, factor graphs were additionally employed in sum-product algorithms for efficient marginalization. In the field of robotics and navigation, factor graphs have been widely adopted for solving Simultaneous Localization and Mapping (SLAM) problems, offering a flexible and robust framework for trajectory estimation [16,17]. Further advancements have expanded their applicability to nonlinear constraints and real-time navigation systems [16,18]. Factor graphs have found broad application across numerous fields, serving as a robust framework for localization and trajectory estimation tasks [19,20,21]. Their ability to integrate all available sensor measurements in a batch optimization setting makes them particularly well-suited for environments with complex motion dynamics and sparse observations. Despite their proven advantages, the application of factor graphs for tracking handheld GPR devices during terrain scans remains a relatively underexplored area, motivating this study.

In our previous research [10,11], a system that processes distance measurements between stationary and mobile ultrawideband (UWB) radio transceivers using various extended Kalman filters (EKFs) was detailed. Building on that foundation, we now present a factor-graph-based estimation algorithm designed to further enhance the accuracy of HH-GPR antenna trajectory estimation. This article aims to investigate the use of factor graphs as an alternative to traditional filtering techniques for estimating the position and trajectory of a handheld GPR system during terrain scanning. The main contributions of this paper are as follows:

1. A comprehensive development and detailed presentation of a factor-graph-based algorithm for HH-GPR antenna trajectory estimation, including analysis of its computational complexity and memory usage, with a comparison to EKF. The proposed algorithm is used to process distance measurements in a UWB positioning system and uses two variants of motion models and two variants of observation models.
2. Presentation of new results of simulative tests for the UWB positioning system with the factor-graph-based algorithm and their comparison with the results obtained with the use of the EKF.

The reminder of this paper is organized as follows. A description of the UWB positioning system and the formulation of the localization problem within the factor graph framework are given in Section 2. The methodology and the results of simulative testing of the factor graph algorithm as well as its comparison against the EKF are presented in Section 3. Finally, Section 4 contains concluding remarks.

## 2. Positioning System and Estimation Algorithm

### 2.1. Positioning System for Handheld Ground-Penetrating Radar

**Figure 1:**
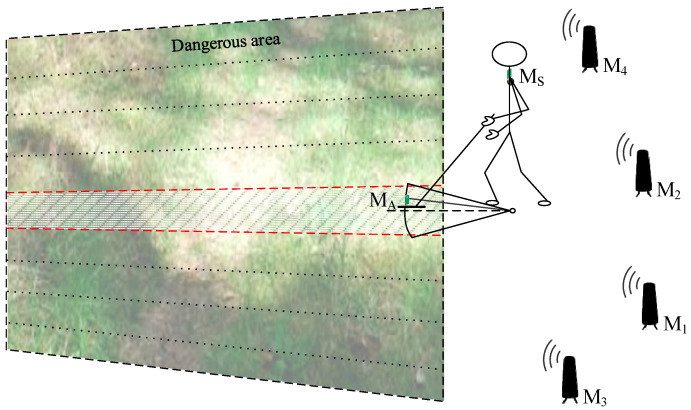
Scanning successive strips of terrain within a zone of predefined boundaries.

**Figure 2:**
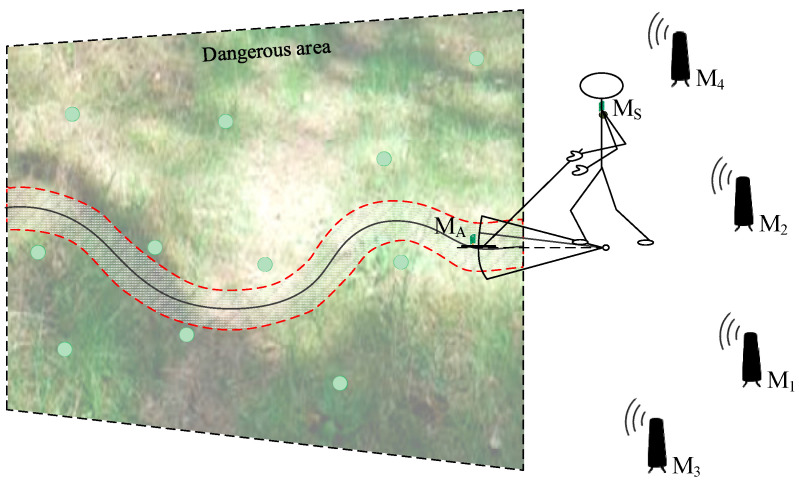
Ad hoc scanning to determine a safe route through a hazardous zone.

In the practical application of a handheld ground-penetrating radar, two operational approaches can be considered. The first approach involves conducting the scanning process over a predefined area—for example, when searching a hazardous region with clearly established boundaries. In such cases, the area may be divided into strips of appropriate width, which are then scanned sequentially as shown in Figure 1. An alternative scenario involves performing ad hoc scanning in dangerous, hard-to-access terrain along the operator’s irregular path, such as when determining a safe route through a hazardous zone, as explained in Figure 2. Both figures also include components of the positioning system, which will be described in detail later in the paper.

**Figure 3:**
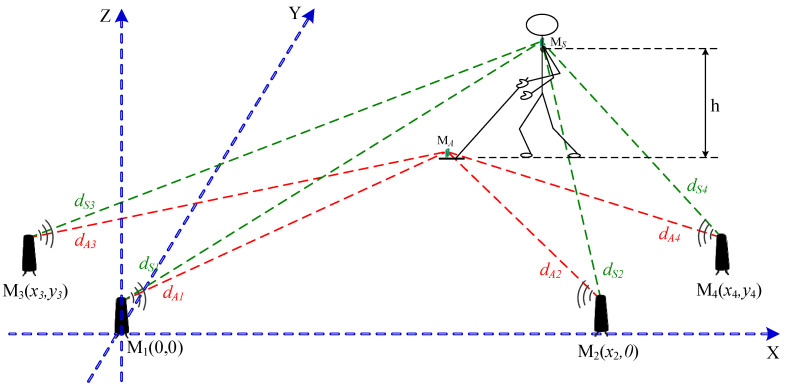
UWB positioning system for HH-GPR.

For these defined scenarios, we have developed a positioning system that utilizes distance measurements between stationary and mobile ultrawideband (UWB) radio transceivers. The system, shown in Figure 3 and described in detail in [10,11], is presented here briefly for completeness. It comprises four UWB base stations of known locations (labeled $M_{1}\;÷\;M_{4}$), strategically positioned in a safe area outside the designated scanning region, and two mobile UWB units: $M_{A}$, mounted on the GPR antenna, and $M_{S}$, attached to the shoulder of the sapper.

The measurement system operates by simultaneously determining the distances between each base station and the mobile units ($d_{A1}\;÷\;d_{A4}$ and $d_{S1}\;÷{\;d}_{S4}$). In our previous works [10,11], to process these measurements and estimate the HH-GPR antenna’s position along with other motion parameters, we applied various custom estimation algorithms based on the Kalman filter. Several dynamic models—including a constant velocity (CV) model and a proprietary pendulum model (PND)—were employed. Although these methods significantly reduced positioning errors compared to a UWB system without filtration, achieving centimeter-level accuracy, the factor-graph-based algorithm discussed in this paper appears to be a promising step forward for even better estimation results.

### 2.2. Application of Factor Graph in Positioning of Handheld Ground-Penetrating Radar

A factor graph is a type of bipartite graph that provides a structured way to model problems involving two distinct types of nodes: hidden variables and factors. Variable nodes correspond to the key unknowns that need to be estimated, such as the state trajectory of an object, while factor nodes impose constraints (such as dynamic constraints), or incorporate measurements that relate to these variables.

In this framework, connections exist only between variable nodes and factor nodes, indicating dependencies between them. An edge linking a variable node to a factor node signifies that the variable’s value influences the factor’s computation or plays a role in determining its outcome. By structuring problems in this manner, a complex global function can be broken down into a collection of simpler local functions, making analysis and computation more efficient.

**Figure 4:**
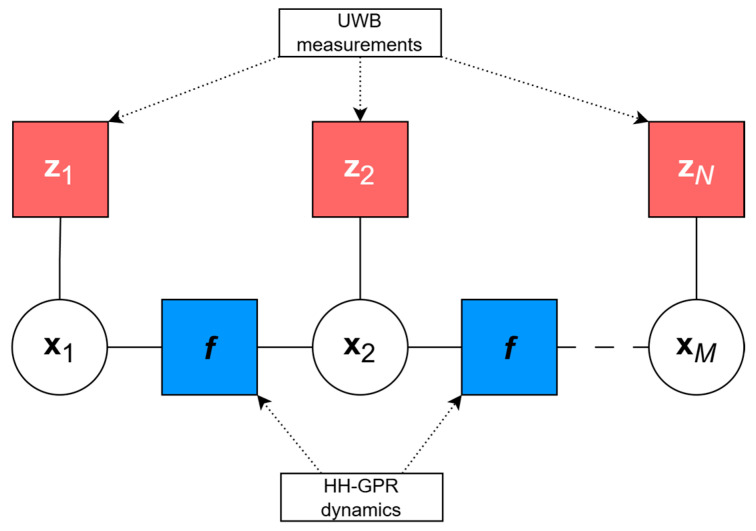
The graph representing trajectory estimation problem without pseudo-measurement.

**Figure 5:**
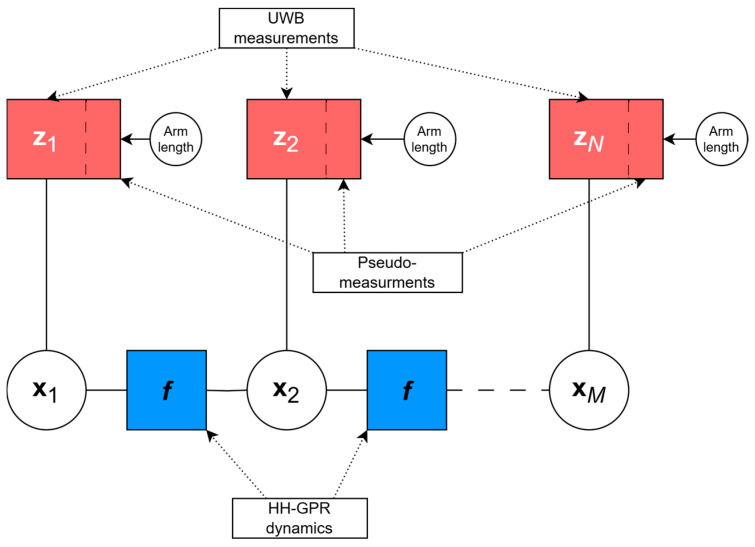
The graph representing trajectory estimation problem with pseudo-measurement.

The factor graph describing the analyzed problem of estimating the HH-GPR position using UWB modules consists of:

- Variable nodes representing the GPR antenna’s state at different time steps ($x_{1}$…$x_{M}$), which include its position and velocity with accordance to a given motion model.
- Factor nodes, which either represent likelihood terms derived from range measurements ($z_{1}\;$…$\;z_{N}$, shown in red in Figure 4 and Figure 5) or transition model functions (f, shown in blue in Figure 4 and Figure 5) that encode dynamic behavior of the system.

The conducted research considered and compared factor graphs constructed for two variants of motion models and two observation models. The motion models included the Constant Velocity (CV) model [10,12]:

(1) $$\left[\begin{array}{c} {\dot{x}}_{A}\\ {\dot{v}}_{x}\\ {\dot{y}}_{A}\\ {\dot{v}}_{y} \end{array}\right]=\left[\begin{array}{cccc} 0 & 1 & 0 & 0\\ 0 & 0 & 0 & 0\\ 0 & 0 & 0 & 1\\ 0 & 0 & 0 & 0 \end{array}\right]\left[\begin{array}{c} x_{A}\\ v_{x}\\ y_{A}\\ v_{y} \end{array}\right]+\left[\begin{array}{cc} 0 & 0\\ 1 & 0\\ 0 & 0\\ 0 & 1 \end{array}\right]\left[\begin{array}{c} u_{v_{x}}\\ u_{v_{y}} \end{array}\right],$$

and a proprietary non-linear model based on the pendulum equation (PND) [10,11]:

(2) $$\left[\begin{array}{c} {\dot{x}}_{A}\\ {\dot{y}}_{A}\\ {\dot{x}}_{S}\\ {\dot{y}}_{S}\\ \dot{\theta }\\ \dot{\omega }\\ \dot{a} \end{array}\right]=\left[\begin{array}{c} \omega \left(y_{A}-y_{S}\right)\\ -\omega \left(x_{A}-x_{S}\right)\\ 0\\ 0\\ \omega \\ -\frac{a}{l}\sin\theta \\ 0 \end{array}\right]+\left[\begin{array}{ccc} 1 & 0 & 0\\ 0 & 1 & 0\\ 1 & 0 & 0\\ 0 & 1 & 0\\ 0 & 0 & 0\\ 0 & 0 & 0\\ 0 & 0 & 1 \end{array}\right]\left[\begin{array}{c} u_{x_{S}}\\ u_{y_{S}}\\ u_{a} \end{array}\right],$$

where:

$x_{A},y_{A}$—coordinates of the $M_{A}$ module position,

$v_{x},v_{y}$—orthogonal velocities of the $M_{A}$ module position,

$x_{S},y_{S}$—coordinates of the $M_{S}$ module position,

$u_{x_{S}},u_{y_{S}}$—Gaussian white noises modeling random $M_{S}$ module motions,

l—horizontal distance between $M_{S}$ and $M_{A}$,

θ—angle between the horizontal projection of the HH-GPR antenna handle and the central axis of the scanning section,

ω—angular velocity of the $M_{A}$ module motion,

a—acceleration forcing the $M_{A}$ module motion, and

$u_{a}$—Gaussian white noise representing random changes of a.

Both observation models used eight distance measurements between each base station and the mobile units ($d_{A1}÷d_{A4}$ and $d_{S1}÷d_{S4}$). They differed in whether a pseudo-measurement for the arm length l was included—a parameter that remains nearly constant and depends on both the HH-GPR handle length and the sapper’s arm height h. The version without this pseudo-measurement is presented in Equation (3).

(3) $$\left[\begin{array}{c} d_{A1}\left(k\right)\\ d_{A2}\left(k\right)\\ d_{A3}\left(k\right)\\ d_{A4}\left(k\right)\\ d_{S1}\left(k\right)\\ d_{S2}\left(k\right)\\ d_{S3}\left(k\right)\\ d_{S4}\left(k\right) \end{array}\right]=\left[\begin{array}{c} \sqrt{{\left(x_{A}\left(k\right)-x_{1}\right)}^{2}+{\left(y_{A}\left(k\right)-y_{1}\right)}^{2}}\\ \sqrt{{\left(x_{A}\left(k\right)-x_{2}\right)}^{2}+{\left(y_{A}\left(k\right)-y_{2}\right)}^{2}}\\ \sqrt{{\left(x_{A}\left(k\right)-x_{3}\right)}^{2}+{\left(y_{A}\left(k\right)-y_{3}\right)}^{2}}\\ \sqrt{\begin{array}{c} {\left(x_{A}\left(k\right)-x_{4}\right)}^{2}+{\left(y_{A}\left(k\right)-y_{4}\right)}^{2} \end{array}}\\ \sqrt{{\left(x_{S}\left(k\right)-x_{1}\right)}^{2}+{\left(y_{S}\left(k\right)-y_{1}\right)}^{2}+h^{2}}\\ \sqrt{{\left(x_{S}\left(k\right)-x_{2}\right)}^{2}+{\left(y_{S}\left(k\right)-y_{2}\right)}^{2}+h^{2}}\\ \sqrt{{\left(x_{S}\left(k\right)-x_{3}\right)}^{2}+{\left(y_{S}\left(k\right)-y_{3}\right)}^{2}{+h}^{2}}\\ \sqrt{\begin{array}{c} {\left(x_{S}\left(k\right)-x_{4}\right)}^{2}+{\left(y_{S}\left(k\right)-y_{4}\right)}^{2}+h^{2} \end{array}} \end{array}\right]+\left[\begin{array}{c} v_{A1}\left(k\right)\\ v_{A2}\left(k\right)\\ v_{A3}\left(k\right)\\ v_{A4}\left(k\right)\\ v_{S1}\left(k\right)\\ v_{S2}\left(k\right)\\ v_{S3}\left(k\right)\\ v_{S4}\left(k\right) \end{array}\right],$$

where:

$d_{Aj}$—distance between a j-th beacon and the $M_{A}$ module,

$d_{Sj}$—distance between a j-th beacon and the $M_{S}$ module,

$x_{j},y_{j}$—coordinates of a j-th beacon position,

$x_{A},y_{A}$—coordinates of the $M_{A}$ module position,

$x_{S},y_{S}$—coordinates of the $M_{S}$ module position,

h—sapper’s arm height, and

$v_{Aj},v_{Sj}$—distance measuring errors for $M_{A}$ and $M_{S}$ modules.

Figure 4 illustrates a factor graph used to estimate the trajectory of a handheld ground-penetrating radar antenna, based on the observation model presented in Equation (3). Each factor represents a distinct element of the trajectory, such as state transitions (motion model) or alignment with UWB observations (measurement model).

When a pseudo-measurement accounting for the length l is included, the observation model presented in Equation (3) is augmented by appending the following equation, which represents a system constraint:

(4) $$l=\sqrt{\begin{array}{c} {\left(x_{S}\left(k\right)-x_{A}\left(k\right)\right)}^{2}+{\left(y_{S}\left(k\right)-y_{A}\left(k\right)\right)}^{2} \end{array}}+v_{SA}\left(k\right),$$

where $v_{SA}$ represents the pseudo-measurement error.

The factor graph for the system model incorporating Equation (4) is illustrated in Figure 5.

### 2.3. Factor-Graph-Based Estimation Algorithm Details

A factor graph represents the joint probability distribution of a set of variables by expressing it as a product of multiple factors. This formulation allows the problem to be reinterpreted as an optimization task, where the objective is to minimize the sum of residuals associated with all factors.

For trajectory estimation, this optimization problem can be written as:

(5) $$\begin{array}{r} \hat{\chi }=\arg\underset{\chi }{\min}\sum_{i=1}^{M}\;{\left(f\left(x_{i-1}\right)-x_{i}\right)}^{T}Q^{-1}\left(f\left(x_{i-1}\right)-x_{i}\right)+\\ +\sum_{k=1}^{N}\;{\left(h\left(x_{k}\right)-z_{k}\right)}^{T}R^{-1}\left(h\left(x_{k}\right)-z_{k}\right), \end{array}$$

where χ represents the set of possible states, M is the number of dynamic constraints along trajectory, f denotes the transition vector function which describes system dynamics, $x_{i}$ is the state vector at i-th step, Q is the process noise covariance matrix, N is the number of measurements, h represents the measurement function, $z_{k}$ is the k-th measurement and R denotes the covariance matrix for measurement noise. Equation (5) can be simplified using the ${\left\|\cdot \right\|}_{\Sigma }^{2}$ notation for a squared Mahalanobis distance, given a covariance matrix Σ:

(6) $$\hat{\chi }=\arg\underset{\chi }{\min}\sum_{i=1}^{M}\;{\left\|f\left(x_{i-1}\right)-x_{i}\right\|}_{Q}^{2}+\sum_{k=1}^{N}\;{\left\|h\left(x_{k}\right)-z_{k}\right\|}_{R}^{2}.$$

Solving this optimization problem involves determining the state configuration that minimizes the negative log-likelihood via a weighted least-squares approach. This is achieved through error minimization using iterative local linearization with either Gauss–Newton or Levenberg–Marquardt methods. Terms in the least-squares objective function (6) associated with factors defined by f(x) and h(x) are approximated using a Taylor expansion around the current estimate $\hat{x}$. To this end, the following relationships can be applied:

(7) $$\begin{array}{c} {\left(f\left(x_{i-1}+{∆x}_{i-1}\right)-\left(x_{i}+{∆x}_{i}\right)\right)}^{T}Q^{-1}\left(f\left(x_{i-1}+{∆x}_{i-1}\right)-\left(x_{i}+{∆x}_{i}\right)\right)=\\ ={\left(f\left(x_{i-1}\right)-x_{i}+\left[\begin{array}{cc} F_{i-1} & -I \end{array}\right]\left[\begin{array}{c} {∆x}_{i-1}\\ {∆x}_{i} \end{array}\right]\right)}^{T}Q^{-1}\left(f\left(x_{i-1}\right)-x_{i}+\left[\begin{array}{cc} F_{i-1} & -I \end{array}\right]\left[\begin{array}{c} {∆x}_{i-1}\\ {∆x}_{i} \end{array}\right]\right)=\\ {=\left\|f\left(x_{i-1}\right)-x_{i}+\left[\begin{array}{cc} F_{i-1} & -I \end{array}\right]\left[\begin{array}{c} {∆x}_{i-1}\\ {∆x}_{i} \end{array}\right]\right\|}_{Q}^{2}, \end{array}$$

(8) $$\begin{array}{c} {\left(h\left(x_{k}+∆x_{k}\right)-z_{k}\right)}^{T}R^{-1}\left(h\left(x_{k}+∆x_{k}\right)-z_{k}\right)=\\ ={\left(h\left(x_{k}\right)-z_{k}+H_{k}∆x_{k}\right)}^{T}R^{-1}\left(h\left(x_{k}\right)-z_{k}+H_{k}∆x_{k}\right)=\\ ={\left\|h\left(x_{k}\right)-z_{k}+H_{k}∆x_{k}\right\|}_{R}^{2}, \end{array}$$

where $F_{i-1}$ is a Jacobian of $f\left(x_{i-1}\right)$ and $H_{k}$ is a Jacobian of $h\left(x_{k}\right)$. To simplify the notation of further calculations, we replace the functions $f\left(x_{i-1}+{∆x}_{i-1}\right)-\left(x_{i}+{∆x}_{i}\right)$ and $h\left(x_{k}+∆x_{k}\right)-z_{k}$ with a unified error function e(x) with its covariance matrix **Σ**. Accounting for the M dynamic constraints and N measurement factors, Equation (6) becomes:

(9) $$\hat{\chi }=\arg\underset{\chi }{\min}\sum_{j=1}^{M+N}\;{\left\|e_{j}(x)\right\|}_{\Sigma }^{2},$$

This can be rewritten as searching for the optimal step:

(10) $$\Delta \hat{\chi }=\arg\underset{\Delta \chi }{\min}\sum_{j=1}^{M+N}\;{\left\|e_{j}(x+\Delta x)\right\|}_{\Sigma }^{2},$$

Which can then provide state estimates that are incrementally refined through iterative updates:

(11) $${\hat{\chi }}_{j}={\hat{\chi }}_{j-1}+\Delta {\hat{\chi }}_{j}.$$

One approach to solving this optimization problem is to apply QR decomposition [18]. To this end, the appropriate transformations of Equation (10) must be performed based on putting the Mahalanobis distance in terms of the Euclidean distance with $\Sigma^{1/2}$ as the matrix square root of Σ:

(12) $$\;{\left\|e_{j}(x)\right\|}_{\Sigma }^{2}={\left\|\Sigma^{-T/2}e_{j}(x)\right\|}^{2},$$

Leading to restating the optimal step search as:

(13) $$\Delta \hat{\chi }=\arg\underset{\Delta \chi }{\min}\sum_{j=1}^{M+N}\;{\left\|{\Sigma_{j}}^{-T/2}e_{j}(x)+{\Sigma_{j}}^{-T/2}J_{j}\Delta x\right\|}^{2},$$

where $J_{j}$ is the Jacobian of the j-th error function.

By defining two new structures, a sparse matrix A:

(14) $$A=\left[\begin{array}{ccc} {\Sigma_{1}}^{-T/2}J_{1} & \ldots & \ldots \\ \vdots & {\Sigma_{j}}^{-T/2}J_{j} & \vdots \\ \ldots & \ldots & {\Sigma_{M+N}}^{-T/2}J_{M+N} \end{array}\right],$$

and a stacked vector b:

(15) $$b=\left[\begin{array}{c} {\Sigma_{1}}^{-T/2}e_{1}(x)\\ \begin{array}{c} \vdots \\ {\Sigma_{j}}^{-T/2}e_{j}(x)\\ \vdots \end{array}\\ {\Sigma_{M+N}}^{-T/2}e_{M+N}(x) \end{array}\right],$$

and using A and b, the optimization criterion can be formulated as a typical least-squares problem:

(16) $$\Delta \hat{\chi }=\arg\underset{\Delta \chi }{\min}\;{\left\|A\Delta \chi +b\right\|}^{2}.$$

**Figure 6:**
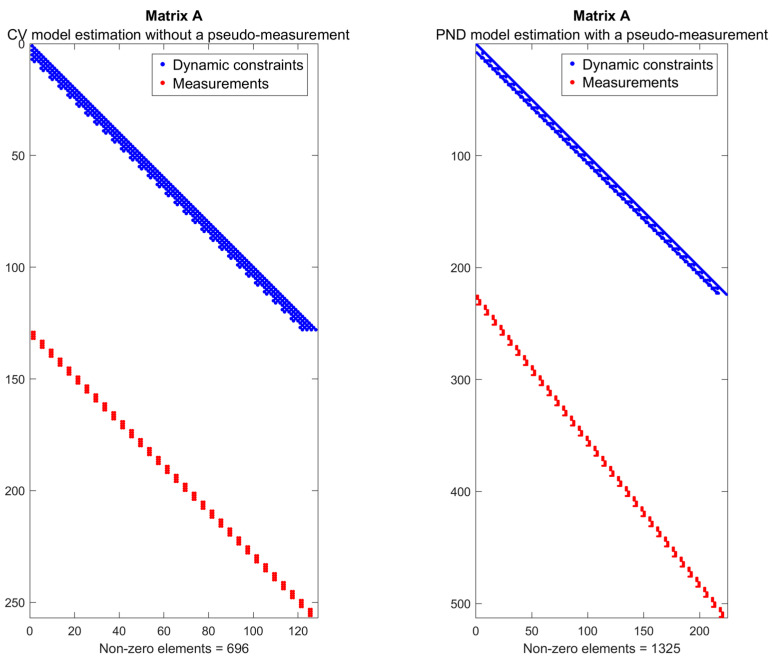
Sparse matrices for optimization problem (**left**: constant velocity motion model without pseudo-measurements, **right**: pendulum motion model with pseudo-measurements).

The above derivation interprets the factor graph problem as a sparse matrix representation, reformulating the estimation process as the iterative solution of a system of linear equations. Here, A is the sparse information matrix of the system [18], $\Delta \hat{\chi }$ represents the update to the state variables, and b is a residual vector containing stacked residuals from all constraints and measurements. Since factor graphs typically exhibit sparse interactions—with each variable influenced by only a limited number of factors—the matrix A is structured so that each hidden variable corresponds to a block of columns, and each factor corresponds to a block of rows, with nonzero entries appearing only where a variable and a factor are directly connected. To demonstrate the sparsity of the A matrix in the factor-graph algorithm, an example from one simulation is shown in Figure 6.

The above figure illustrates the matrix structure, where each block corresponds to a different type of constraint. Such sparsity, resulting from the limited interactions among state variables, enables efficient numerical solutions. Blue elements represent dynamic constraints linking consecutive states, while red elements denote measurement factors. Understanding this structure is essential, as it allows the use of numerical solvers that exploit sparsity to improve computational efficiency.

The solution for the optimization problem stated in Equation (16) can be found by transforming the matrix A using QR decomposition:

(17) $$A=Q_{qr}\left[\begin{array}{c} R_{qr}\\ 0 \end{array}\right],$$

where $Q_{qr}$ is a rotation matrix and $R_{qr}$ is an upper-triangular matrix which is $A^{T}A={R_{qr}}^{T}R_{qr}$. In the context of sparse factor graph optimization, the QR decomposition is performed using implicit transformations (e.g., Givens rotations or Householder reflections) [19], and the explicit form of the rotation matrix $Q_{qr}$ is typically not constructed at all as it is not necessary in further calculations. Instead, the focus in these algorithms is placed on the resulting upper-triangular structure $R_{qr}$. In our experiments, for the QR decomposition we rely on MATLAB^®^ version 2024b implementation of SuiteSparseQR [22], which is designed to handle sparse matrices effectively.

Using Equation (17) the following derivation is performed:

(18) $$\;{\left\|A\Delta \chi +b\right\|}^{2}={\left\|Q_{qr}\left[\begin{array}{c} R_{qr}\\ 0 \end{array}\right]\Delta \chi +b\right\|}^{2}.$$

The $Q_{qr}$ matrix and consequently ${Q_{qr}}^{T}$ are orthogonal matrices, so ${Q_{qr}}^{T}$ can be applied to the right-hand side of Equation (18). As $det\;{Q_{qr}}^{T}=1$, it preserves the Euclidean norm:

(19) $${\left\|Q_{qr}\left[\begin{array}{c} R_{qr}\\ 0 \end{array}\right]\Delta \chi +b\right\|}^{2}={\left\|{{Q_{qr}}^{T}Q}_{qr}\left[\begin{array}{c} R_{qr}\\ 0 \end{array}\right]\Delta \chi +{Q_{qr}}^{T}b\right\|}^{2},$$

and then, as ${{Q_{qr}}^{T}Q}_{qr}=I$, the right-hand side of Equation (19) can be further simplified as follows:

(20) $${\left\|{{Q_{qr}}^{T}Q}_{qr}\left[\begin{array}{c} R_{qr}\\ 0 \end{array}\right]\Delta \chi +{Q_{qr}}^{T}b\right\|}^{2}={\left\|\left[\begin{array}{c} R_{qr}\\ 0 \end{array}\right]\Delta \chi +{Q_{qr}}^{T}b\right\|}^{2}.$$

By introducing the partition ${Q_{qr}}^{T}b=\left[\begin{array}{c} d\\ c \end{array}\right]$, where the vector d $\in R^{Mn_{x}}$ corresponds to the part matching the size of Δχ and $c\in R^{Nn_{z}}$ is the remaining part, the norm can be split accordingly, leading to the final simplification:

(21) $${\left\|\left[\begin{array}{c} R_{qr}\\ 0 \end{array}\right]\Delta \chi +{Q_{qr}}^{T}b\right\|}^{2}={\left\|\left[\begin{array}{c} R_{qr}\\ 0 \end{array}\right]\Delta \chi +\left[\begin{array}{c} d\\ c \end{array}\right]\right\|}^{2}={\left\|R_{qr}\Delta \chi +d\right\|}^{2}+{\left\|c\right\|}^{2}.$$

Since ${\left\|c\right\|}^{2}$ is a constant, it does not depend on Δχ and the minimization problems reduces to solving:

(22) $$R_{qr}\Delta \chi =-d,$$

which is an upper-triangular system that can be solved efficiently using back-substitution [18]. Once a solution $\Delta \hat{\chi }$ is obtained, the state vector $\hat{\chi }$ is updated according to the iterative procedure from Equation (11). The algorithm based on QR factorization is more numerically stable than solving the normal equations $A^{T}A\Delta \hat{\chi }=A^{T}b$ [18].

### 2.4. Computational Complexity and Memory Usage

From a computational standpoint, the extended Kalman filter and factor graph-based optimization represent fundamentally different approaches to state estimation. EKF is a recursive filter that proceeds over M prediction steps and N correction steps (which often can be equal). The prediction step typically involves a matrix multiplication and covariance propagation with a computational cost of $O\left(n_{x}^{2}\right)$, where $n_{x}$ is the length of the state vector. The update step requires inverting the dense innovation covariance matrix which is the most computationally intensive operation with an asymptotic complexity of $O\left(n_{z}^{2.8}\right)$, where $n_{z}$ is the length of the measurement vector [16]. Thus, the total computational cost of EKF can be approximated as $O\left({Mn}_{x}^{2}+{Nn}_{z}^{2.8}\right)$. In terms of memory, EKF requires maintaining and updating a state vector with $n_{x}$ elements and a dense covariance matrix of size $n_{x}\times n_{x}$.

In contrast, factor graph-based methods formulate the estimation problem as a global non-linear least squares optimization, typically solved over L iterations using iterative linearization techniques. In each iteration, a sparse linear system AΔχ+b is constructed at a cost of $O(Mn_{x}+Nn_{z})$. The system is than solved for $\Delta \hat{\chi }$ using the sparse QR factorization which is $O(\left(Mn_{x}+Nn_{z}\right)log\left(Mn_{x}\right))$ and this leads to the following total asymptotic complexity of the factor graph method $O(L\left(Mn_{x}+Nn_{z}\right)log\left(Mn_{x}\right))$ [23]. The batch factor graph approach requires significantly more memory than EKF—due to the need to store the matrix A and vector b which have sizes $\left(Mn_{x}+Nn_{z}\right)\times Mn_{x}$ and $\left(Mn_{x}+Nn_{z}\right)\times 1$ respectively. It is worth mentioning that by applying fixed-lag smoothing or sliding window techniques, both computational complexity and memory usage can be bounded, thereby enabling the practical deployment of factor graph-based optimization on embedded platforms [18].

In summary, EKF offers low computational complexity and memory footprint per iteration, making it suitable for real-time, resource-constrained applications. However, factor graph optimization provides higher accuracy and scalability, particularly in large-scale or high-precision systems, albeit at the cost of increased computational and memory requirements.

## 3. Results

**Figure 7:**
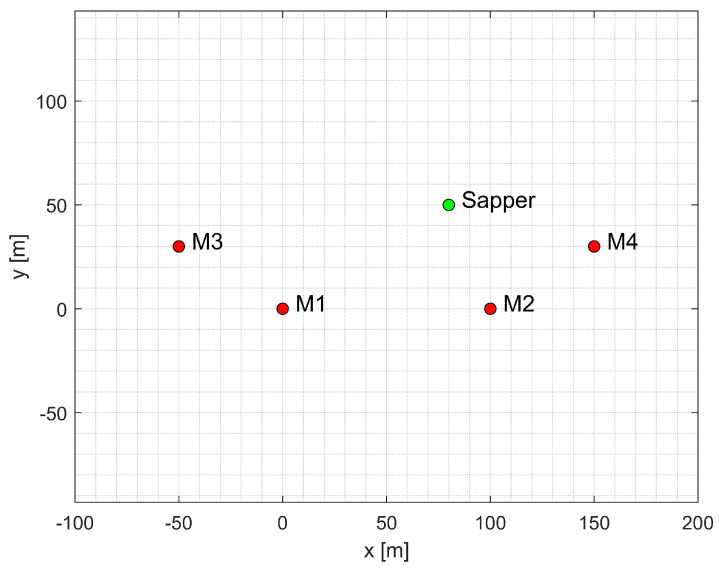
Locations of the base stations and the sapper for the assumed experiment scenario.

The described UWB positioning system dedicated for HH-GPR was modelled in MATLAB^®^ version 2024b. The simulation of its operation and the demonstration of its performance using various estimation algorithms required several steps. First, assumptions regarding the layout of the positioning system’s components were adopted. The assumed locations of the base stations and the sapper were as follows: $M_{1}\left(0,0\right)$, $M_{2}\left(100,0\right)$, $M_{3}\left(-50,30\right)$, $M_{4}\left(150,30\right)$, and $M_{S}\left(80,50\right)$, and they are presented in Figure 7.

Then, the GPR antenna trajectory was generated, assuming similar motion parameters as described in our previous work [11]. Similarly to [11], the measurements between pairs of mobile UWB modules $M_{S}$ and $M_{A}$ and stationary modules $M_{1}{÷M}_{4}$ were generated assuming that their errors are normally distributed with zero mean and a standard deviation of 2 cm. These measurements were further processed by various filters.

All the considered estimation algorithms were implemented. They included the extended Kalman filter with the PND dynamics model, which proved to be the most accurate version of the EKF in our previous research [11], and four versions of factor-graph algorithms. The factor-graph algorithms differed in the dynamics model used (CV or PND) and in whether a pseudo-measurement for the arm length was included. The parameters assumed in the dynamics and observation models underlying both the EKF and factor-graph algorithms were chosen, as in [11], to tune the algorithms and minimize their estimation errors.

**Figure 8:**
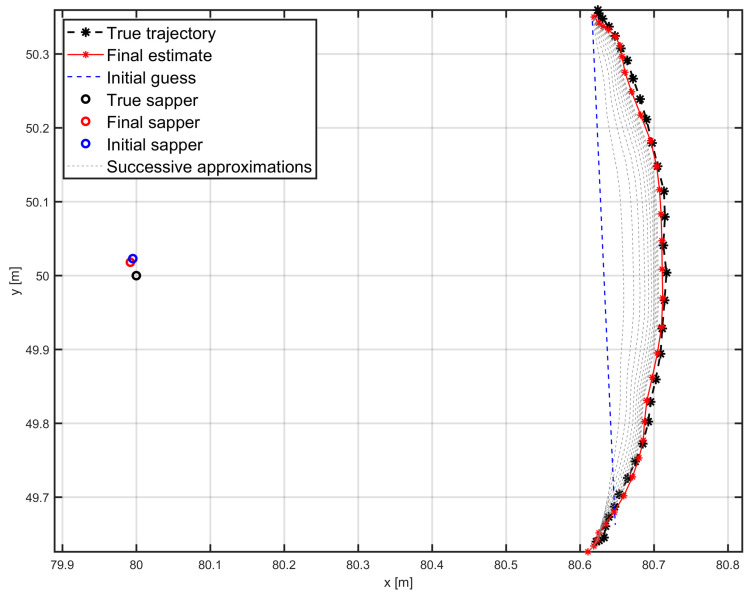
HH-GPR antenna trajectory estimation using factor graph algorithm with CV dynamics model and pseudo-measurements.

Based on these assumptions, multiple simulation runs were performed and the estimated position of the HH-GPR antenna was acquired. Based on these results, the average RMS errors along the entire trajectory were calculated. Example results of trajectory estimation using a factor graph with the CV motion model and with pseudo-measurements are shown in Figure 8. This figure illustrates how the estimated trajectory evolves from its initial guess, through successive approximations, to the final smooth result.

**Figure 9:**
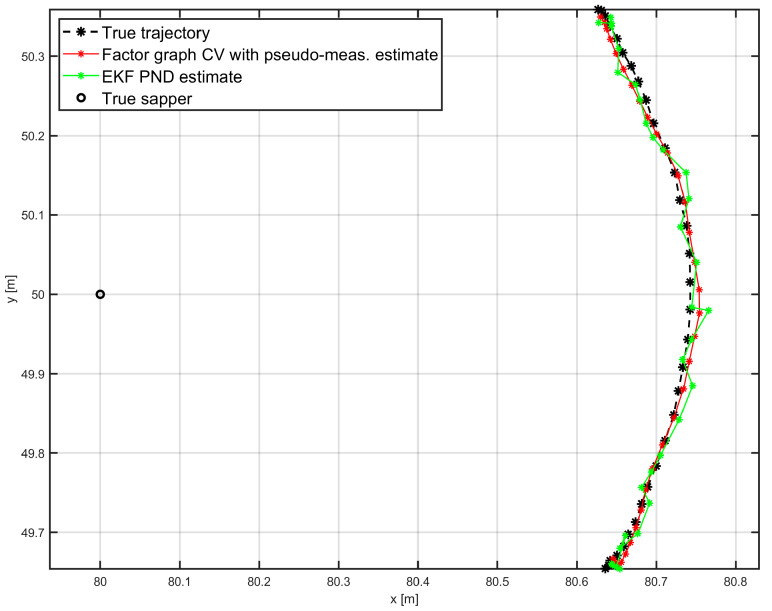
HH-GPR antenna estimation comparison for factor graph algorithm with CV dynamics model and pseudo-measurements and EKF with PND dynamics model.

Figure 9 compares the final estimated trajectory from the factor-graph algorithm with the trajectory estimated using the EKF with the PND model. This version of the EKF proved to be superior to other extended Kalman filters in our previous research [10,11]. Although the factor-graph algorithm shows even better accuracy in this single-run demonstration, this result is provided solely for illustration purposes.

A more comprehensive comparison of the positioning accuracy of the various considered algorithms is provided in Table 1. These results are based on 300 simulation runs with different realizations of process noise and measurement errors.

As can be seen, all the factor-graph algorithms are more accurate than the best-performing EKF based on the pendulum model. The improvement in accuracy ranges from over 30 to almost 50 percent, which paves the way for enhanced performance of the positioning system. This improved accuracy in practical GPR applications implies that the synthesized underground images may be less blurred and more distinct, thereby facilitating the identification and classification of detected objects and infrastructure.

## 4. Conclusions

The simulation results demonstrate that factor graph-based estimation provides higher accuracy in determining the position of the handheld ground-penetrating radar (HH-GPR) antenna compared to the extended Kalman filter. As previously mentioned, EKF operates as a Markov process, which can introduce estimation inconsistencies over time, whereas factor graphs leverage the entire trajectory, resulting in a smoother and more precise solution.

Among the evaluated factor graph models, the one incorporating the dedicated PND dynamics outperformed the model based on the simpler CV assumption. This indicates that for HH-GPR motion, a more complex dynamic model can provide better estimation accuracy, likely due to its ability to capture the specific characteristics of the sapper’s movement. Nevertheless, the CV model remains attractive due to its simplicity and lower computational complexity—it avoids the need to compute Jacobians at every iteration and for every factor, making it well-suited for applications with limited processing power. Furthermore, the inclusion of a pseudo-measurement—specifically, the known length of the sapper’s arm—further enhanced the performance, highlighting the value of integrating prior structural constraints into the estimation framework.

An additional advantage of the factor graph approach is its flexibility in incorporating modifications. The framework allows for easy integration of alternative motion models or additional pseudo-measurements without fundamentally changing the estimation structure. This adaptability makes factor graphs a robust tool for refining and optimizing estimation accuracy in various operational conditions.

While these findings provide valuable insights into antenna position estimation for HH-GPR, future work will focus on validating the proposed methods in real-world conditions. Planned experiments will involve high-precision reference systems such as Vicon to obtain ground-truth trajectories and assess the practical performance of the factor graph-based approach. These real-world evaluations will help refine the estimation models and further confirm the advantages of factor graph optimization over traditional filtering methods.

## Figures and Tables

**Table 1:** Comparison of RMS positioning errors for tested estimation algorithms.

Estimation Method	RMSE [m]	Improvement [%]
EKF PND	0.0149	-
Factor graph (CV)	0.0097	34.9
Factor graph (CV + pseudo-measurement)	0.0091	39.0
Factor graph (PND)	0.0080	46.3
Factor graph (PND + pseudo-measurement)	0.0076	49.0

## Data Availability

The raw data supporting the conclusions of this article will be made available by the authors on request.

## Abbreviations

The following abbreviations are used in this manuscript:

HH-GPR	Handheld Ground-Penetrating Radar
EKF	Extended Kalman Filter
PND	Pendulum
KF	Kalman Filter
SLAM	Simultaneous Localization and Mapping
UWB	Ultrawideband
CV	Constant Velocity
